# Spatial navigation intervention informed by region-specific brain activation for individuals at risk for dementia

**DOI:** 10.1093/geroni/igaf122.2273

**Published:** 2025-12-31

**Authors:** Pierfilippo De Sanctis, Sunil Agrawal, Jiawei Chen, Joe Verghese

**Affiliations:** Albert Einstein College of Medicine, Bronx, New York, United States; Columbia University, New York, New York, United States; Columbia University, New York, New York, United States; Stony Brook University, Stony Brook, New York, United States

## Abstract

Up to a third of dementia cases may be preventable by engaging in protective behaviors, such as staying cognitively active, according to observational data. Yet, current cognitive training protocols to delay dementia onset often fall short. This project focuses on spatial navigation (SN), the ability to travel familiar/unfamiliar environments. Tau and amyloid-beta accumulation starts in regions subserving SN. Even though SN difficulties present an important target, there are few clinical trials aimed at SN. We developed a full-immersive virtual-reality (VR) maze which participants learn to navigate and use Mobile Brain Body Imaging (MoBI) to record body movement and EEG to record and analyze brain activity during active movement through space. We designed VR mazes to induce different navigational strategies (allocentric and egocentric) at different periods (Stand/Encode and Walk/Navigate) of maze learning. Allocentric and egocentric spatial strategies rely on mediotemporal and posterior parietal cortex regions, respectively. So far, we collected data in 10 individuals showing that Stand/Encode time increases as maze complexity increases. Furthermore, we show a significant increase in theta power as participants navigate towards an intersection that requires a memory-based directional decision. Region-specific modulations in theta (3-7Hz) and alpha (8-12Hz) power we enable us to identify, dissociate, and track participants’ brain dynamics applying mediotemporal-based allocentric and posterior parietal-based egocentric navigational strategies and test relationships with improvements in SN. This pilot study will position us to design a future randomized clinical trial to test efficacy to improve navigational abilities, and thereby delay cognitive decline in older adults at-risk for dementia.

